# Loss of the cytosolic DNA-sensing genes *CGAS* and *STING1* in armadillos (Cingulata)

**DOI:** 10.1101/2025.05.13.651073

**Published:** 2025-05-16

**Authors:** Lillie Schaffer, Amanda Ivanoff, Frédéric Delsuc, Vincent J. Lynch

**Affiliations:** 1 Department of Biological Sciences, University at Buffalo, SUNY, 551 Cooke Hall, Buffalo, NY, 14260, USA.; 2 Institut des Sciences de l’Evolution de Montpellier (ISEM), CNRS, IRD, Université de Montpellier, Place Eugène Bataillon, 34095 Montpellier, France.

## Abstract

The principal sensor of intracellular double-stranded DNA (dsDNA) is cyclic GMP-AMP synthase (CGAS), which generates the second messenger cyclic GMP-AMP that binds stimulator of interferon genes (STING1), leading to the expression of type I interferon genes. CGAS and STING1 also play essential roles in maintaining genome integrity and the initiation and progression of cancer. Here we show that *CGAS* and *STING1* were pseudogenized in the ancestral armadillo branch 45 to 70 million years ago. The complete loss of the *CGAS*-*STING1* pathway in armadillos suggests this lineage has evolved alternate ways to sense intracellular double-stranded DNA, which may be related to their extreme cancer resistance.

## Introduction

Innate immunity is the first defense against foreign pathogens or endogenous danger signals, with distinct receptors for different danger signals, such as pathogen-associated molecular patterns (PAMPs) or damage-associated molecular patterns (DAMPs), that invoke an intracellular immune response. The principal sensor of intracellular double-stranded DNA (dsDNA) is cyclic GMP-AMP synthase (CGAS). CGAS binds to cytosolic dsDNA, generating the second messenger cyclic GMP-AMP (cGAMP), which then binds to the stimulator of interferon genes (STING1). cGAMP-bound STING subsequently undergoes a conformational change and translocates from the endoplasmic reticulum to the intermediate compartments between the endoplasmic reticulum and Golgi, which recruits TANK-binding kinase-1 (TBK1). TBK1 phosphorylates and activates interferon regulatory factor-3 (IRF3), leading to the expression of type I interferon genes^1^.

## Results

**Figure 1:**
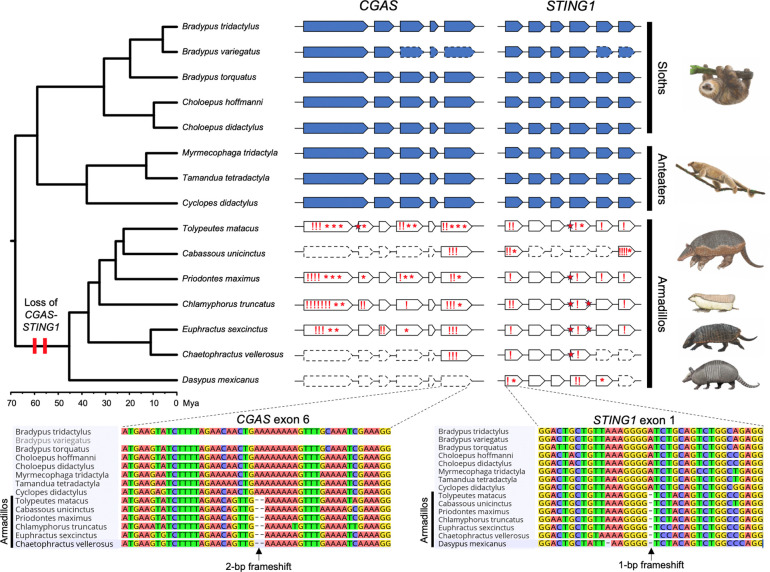
Evolutionary loss of *CGAS-STING1* in armadillos. Genomic organization of *CGAS* and *STING1* exons represented in the phylogenetic context of the 15 investigated xenarthran species (five sloths, three anteaters, and seven armadillos). Functional genes found in sloths and anteaters are represented in blue, and armadillo pseudogenes in white. Dashed line contours show exons missing in current genomic assemblies. Frameshifts (!), stop-codons (*), and splice site mutations (red stars) are indicated in armadillo pseudogenes. Sequence alignments present examples of inactivating mutations in exons shared by all armadillos, indicating that both *CGAS* and *STING1* were lost in their common ancestor 45 to 70 million years ago (Mya). Xenarthran phylogeny and timescale according to Gibb et al. (2016). Paintings by Carl Buell (copyright John Gatesy) and Michelle S. Fabros.

We recently completed a survey of gene losses in *Atlantogenata*^2^ and serendipitously noted that TOGA-based^3^ annotations indicated that the *CGAS* gene was lost in the Southern three-banded armadillo (*Tolypeutes matacus*), Mexican long-nosed armadillo (*Dasypus mexicanus;* formerly *D. novemcinctus*^4^), and giant anteater (*Myrmecophaga tridactyla)*, while *STING1* was lost in the Mexican long-nosed armadillo. To confirm these surprising observations, we used miniprot^5^ and minimap2^6^ to respectively map the Linnaeus’s two-toed sloth (*Choloepus didactylus*) CGAS and STING1 proteins and nucleotide coding sequences to 22 genome assemblies from 15 xenarthran species representing all major lineages (Figure 1). Unlike the TOGA-based annotation, the *CGAS* and *STING1* genes were complete and did not include inactivating mutations in sloths and anteaters (Pilosa). In contrast, *CGAS* had many inactivating mutations in six armadillo species (Cingulata)*,* indicating it was pseudogenized and was completely missing in the chromosome-scale assembly of the Mexican long-nosed armadillo (*D. mexicanus*). *STING1* also had numerous inactivating mutations, including some shared by all seven armadillo species investigated. These data indicate that *CGAS* and *STING1* genes were pseudogenized in the ancestral armadillo branch 45 to 70 million years ago (Mya) (Figure 1). The complete loss of the CGAS-STING1 pathway in a major placental order (Cingulata) is only paralleled by pangolins (Pholidota) in mammals^7^.

## Discussion

CGAS and STING1, widely recognized for their roles in the innate immune system^1^, also play essential roles in maintaining genome integrity and the initiation and progression of cancer. In the nucleus, for example, CGAS binds to chromatin^8–10^ and is recruited to sites of DNA double-strand breaks, where it inhibits RAD51-coated ssDNA filaments from initiating strand invasion^11^ and binds to PARP1 via poly(ADP-ribose), disrupting the formation of the PARP1–Timeless complex^12^. As a result of these processes, homologous recombination is suppressed, leading to increased chromosomal instability (CIN) and promoting tumorigenesis^11,12^. CIN also drives cancer metastasis^13–16^. Remarkably, chronic activation of the CGAS-STING1 pathway due to CIN rewires signaling in cancer cells, creating a pro-metastatic tumor microenvironment^17^. This rewiring causes rapid, diminishing responses (tachyphylaxis) to type-I interferon downstream of STING1; tumor cell-intrinsic STING1 inhibitors reduce CIN-driven metastasis in melanoma, breast, and colorectal cancers^17^.

CGAS and STING1 also play a central role in inflammation and senescence, in which cells with endogenous genomic DNA (gDNA) damage or mitochondrial (mt) dysfunction lead to the release of gDNA or mtDNA into the cytosol, causing activation of the CGAS-STING1 pathway. This can lead to permanent cell cycle arrest (senescence) and the release of inflammatory cytokines, also called the senescence-associated secretory phenotype (SASP)^18^. Prolonged activation of this pathway also leads to chronic systemic inflammation. For example, activation of the CGAS-STING pathway drives aging-related inflammation, neurodegeneration, and cognitive decline^18^. The absence of the CGAS-STING1 cytosolic pathway in armadillos and pangolins suggests that they may have evolved CGAS-STING1-independent mechanisms to respond to intracellular dsDNA, which may contribute to cancer resistance because the cancer-promoting effects of CGAS-STING have also been lost.

## Cancer Prevalence in Armadillos and Pangolins

While armadillos and pangolins do get cancer, it is remarkably rare in armadillos^19^. We compiled previously published data that systematically explore cancer prevalence in armadillos and found only two cases of neoplasia out of 342 necropsy reports, suggesting cancer prevalence is only around 0.58% (Clopper-Pearson exact 95% CI=0.071%–2.09%)^19–23^. An exhaustive literature search identified only a handful of cases of cancer in armadillos, including metastatic squamous cell carcinoma^24^, fibroma^25^, osteosarcoma^26^, adenocarcinoma in the stomach, mammary neoplasia with pulmonary metastasis^27^, a potentially virus-induced primary gastric T-cell lymphoma^28^, bronchiolar carcinoma, and a leiomyoma of the stomach^21^. Cancer can also be experimentally induced in armadillos—thalidomide treatment induced a highly malignant, aggressive choriocarcinoma that metastasized to the liver, mesentery, visceral parietal peritoneum, and lungs; the metastatic tumors in the lungs formed numerous teratomas^29^. In a similarly exhaustive literature search in pangolins, we found sporadic cases of benign hyperplasias, including a hemangioma^30^, an endometrial hyperplasia^31^, bile duct and thyroid gland hyperplasia^32^, and two cases of hepatocellular carcinoma^33^. A larger study of 22 wild-collected Chinese pangolins identified six cases of squamous cell carcinoma, three papillomas, at least one of which had early malignant changes, one case of adenomatoid, and several cases of hyperplasia of the gastric lining in the stomach. Unfortunately, there are no systematic studies in pangolins to reliably estimate their cancer prevalence. Thus, cancer is rare in armadillos, and possibly pangolins, which may be related to the loss of the evolutionary *CGAS* and *STING1* genes.

## Future Directions

The absence of the *CGAS* and *STING1* in armadillos and pangolins suggests that other genes in the pathway may also have been lost, or have altered patterns of molecular evolution, such as relaxed selective constraints or positive selection; characterizing patterns of molecular evolution in armadillos and pangolins might identify such shifts. It is also important to demonstrate that functions of CGAS and STING1 are not compensated by other genes; such inferences will require functional validation.

## Methods

### Inferences of Gene Loss Events

We downloaded TOGA loss_summ_data.tsv files, which used the human hg38 reference for each species, from https://genome.senckenberg.de/download/TOGA/. Gene losses in each species were identified as genes (GENE) annotated as “clearly lost” or “L” and which we coded as state 0, while all other TOGA categories were collapsed into a likely present category and coded as state 1; we note that this coding is conservative, as genes annotated with an “uncertain loss” and “partially intact” may be true losses. However, this coding scheme ensures we only infer losses with the highest confidence of true loss and may miss recent pseudogenization events when there are only one or a few inactivating mutations. Next, we inferred ancestral states and gene losses using the dollop program in Phylip (version 3.695), using the species phylogeny (with Paenungulata lineages as a polytomy) and enforcing all genes as present in the common ancestor; dollop infers ancestral dates using a Dollo’s law of irreversibility, in which once lost a gene cannot reevolve in descent lineages. We note that other processes could lead to an apparent reevolution of a previously lost gene such as incomplete lineage sorting, hybridization, or forms of gene flow such as horizontal gene transfer. While unlikely in mammals, Bayesian implementations of the Dollo model have been developed that account for processes like horizontal transfer^34,35^. To manually confirm these gene losses, we used miniprot^5^ and minimap2^6^ to map the Linnaeus’s two-toed sloth (*Choloepus didactylus*) *CGAS* and *STING1* proteins and nucleotide coding sequences to 22 genome assemblies from 15 xenarthran species representing all major lineages (Table S1). More specifically, miniprot was run with the following parameters: miniprot -j2 --aln --trans --gff to generate residue aligments, CDS predictions, and produce .gff annotation files detailing frameshifts, stop codons, and splice site mutations on predicted exons of each xenarthran assembly. Predicted CDSs were then aligned with MACSEv2^36^ and imported into GeniousPrime 2025.1.1^37^ to confirm frameshifts and stop codons. To further verify and visualize frameshifts and splice site mutations, minmap2 was run in Long-read spliced mode (-ax splice) to map CDSs to xenarthran assemblies within GeneiousPrime.

## Supplementary Material

Supplement 1

## References

[1] Chen Q., Sun L., and Chen Z.J. (2016). Regulation and function of the cGAS-STING pathway of cytosolic DNA sensing. Nat Immunol 17, 1142–1149. 10.1038/ni.3558. 27648547

[2] Birkemeier M., Swindle A., Bowman J., and Lynch V.J. (2024). Pervasive loss of regulated necrotic cell death genes in elephants, hyraxes, and sea cows (Paenungualta). Preprint at bioRxiv, 10.1101/2024.04.04.588129 https://doi.org/10.1101/2024.04.04.588129.

[3] Kirilenko B.M., Munegowda C., Osipova E., Jebb D., Sharma V., Blumer M., Morales A.E., Ahmed A.-W., Kontopoulos D.-G., Hilgers L., (2023). Integrating gene annotation with orthology inference at scale. Science 380, eabn3107. 10.1126/science.abn3107. 37104600 PMC10193443

[4] Barthe M., Rancilhac L., Arteaga M.C., Feijó A., Tilak M.-K., Justy F., Loughry W.J., McDonough C.M., de Thoisy B., Catzeflis F., (2025). Exon Capture Museomics Deciphers the Nine-Banded Armadillo Species Complex and Identifies a New Species Endemic to the Guiana Shield. Syst Biol 74, 177–197. 10.1093/sysbio/syae027. 38907999 PMC11958936

[5] Li H. (2023). Protein-to-genome alignment with miniprot. Bioinformatics 39, btad014. 10.1093/bioinformatics/btad014. 36648328 PMC9869432

[6] Li H. (2018). Minimap2: pairwise alignment for nucleotide sequences. Bioinformatics 34, 3094–3100. 10.1093/bioinformatics/bty191. 29750242 PMC6137996

[7] Fischer H., Tschachler E., and Eckhart L. (2020). Cytosolic DNA sensing through cGAS and STING is inactivated by gene mutations in pangolins. Apoptosis 25, 474–480. 10.1007/s10495-020-01614-4. 32533513 PMC7291609

[8] Kujirai T., Zierhut C., Takizawa Y., Kim R., Negishi L., Uruma N., Hirai S., Funabiki H., and Kurumizaka H. (2020). Structural basis for the inhibition of cGAS by nucleosomes. Science 370, 455–458. 10.1126/science.abd0237. 32912999 PMC7584773

[9] Michalski S., de Oliveira Mann C.C., Stafford C.A., Witte G., Bartho J., Lammens K., Hornung V., and Hopfner K.-P. (2020). Structural basis for sequestration and autoinhibition of cGAS by chromatin. Nature 587, 678–682. 10.1038/s41586-020-2748-0. 32911480

[10] Pathare G.R., Decout A., Glück S., Cavadini S., Makasheva K., Hovius R., Kempf G., Weiss J., Kozicka Z., Guey B., (2020). Structural mechanism of cGAS inhibition by the nucleosome. Nature 587, 668–672. 10.1038/s41586-020-2750-6. 32911482

[11] Jiang H., Xue X., Panda S., Kawale A., Hooy R.M., Liang F., Sohn J., Sung P., and Gekara N.O. (2019). Chromatin-bound cGAS is an inhibitor of DNA repair and hence accelerates genome destabilization and cell death. The EMBO Journal 38, e102718. 10.15252/embj.2019102718. 31544964 PMC6826206

[12] Liu H., Zhang H., Wu X., Ma D., Wu J., Wang L., Jiang Y., Fei Y., Zhu C., Tan R., (2018). Nuclear cGAS suppresses DNA repair and promotes tumorigenesis. Nature 563, 131–136. 10.1038/s41586-018-0629-6. 30356214

[13] Li J., Duran M.A., Dhanota N., Chatila W.K., Bettigole S.E., Kwon J., Sriram R.K., Humphries M.P., Salto-Tellez M., James J.A., (2021). Metastasis and Immune Evasion from Extracellular cGAMP Hydrolysis. Cancer Discov 11, 1212–1227. 10.1158/2159-8290.CD-20-0387. 33372007 PMC8102348

[14] Bakhoum S.F., and Cantley L.C. (2018). The Multifaceted Role of Chromosomal Instability in Cancer and Its Microenvironment. Cell 174, 1347–1360. 10.1016/j.cell.2018.08.027. 30193109 PMC6136429

[15] Bakhoum S.F., Ngo B., Laughney A.M., Cavallo J.-A., Murphy C.J., Ly P., Shah P., Sriram R.K., Watkins T.B.K., Taunk N.K., (2018). Chromosomal instability drives metastasis through a cytosolic DNA response. Nature 553, 467–472. 10.1038/nature25432. 29342134 PMC5785464

[16] Wörmann S.M., Zhang A., Thege F.I., Cowan R.W., Rupani D.N., Wang R., Manning S.L., Gates C., Wu W., Levin-Klein R., (2021). APOBEC3A drives deaminase domain-independent chromosomal instability to promote pancreatic cancer metastasis. Nat Cancer 2, 1338–1356. 10.1038/s43018-021-00268-8. 35121902

[17] Li J., Hubisz M.J., Earlie E.M., Duran M.A., Hong C., Varela A.A., Lettera E., Deyell M., Tavora B., Havel J.J., (2023). Non-cell-autonomous cancer progression from chromosomal instability. Nature 620, 1080–1088. 10.1038/s41586-023-06464-z. 37612508 PMC10468402

[18] Yang H., Wang H., Ren J., Chen Q., and Chen Z.J. (2017). cGAS is essential for cellular senescence. Proc Natl Acad Sci U S A 114, E4612–E4620. 10.1073/pnas.1705499114. 28533362 PMC5468617

[19] Vazquez J.M., Pena M.T., Muhammad B., Kraft M., Adams L.B., and Lynch V.J. (2022). Parallel evolution of reduced cancer risk and tumor suppressor duplications in Xenarthra. Elife 11, e82558. 10.7554/eLife.82558. 36480266 PMC9810328

[20] Bulls S.E., Platner L., Ayub W., Moreno N., Arditi J.-P., Dreyer S., McCain S., Wagner P., Burgstaller S., Davis L.R., (2024). Unraveling the relationship between cancer and life history traits in vertebrates. bioRxiv, 2022.07.12.499088. 10.1101/2022.07.12.499088.

[21] Effron M., Griner L., and Benirschke K. (1977). Nature and rate of neoplasia found in captive wild mammals, birds, and reptiles at necropsy. J Natl Cancer Inst 59, 185–198. 10.1093/jnci/59.1.185. 577508

[22] Vincze O., Colchero F., Lemaître J.-F., Conde D.A., Pavard S., Bieuville M., Urrutia A.O., Ujvari B., Boddy A.M., Maley C.C., (2022). Cancer risk across mammals. Nature 601, 263–267. 10.1038/s41586-021-04224-5. 34937938 PMC8755536

[23] Compton Z.T., Mellon W., Harris V.K., Rupp S., Mallo D., Kapsetaki S.E., Wilmot M., Kennington R., Noble K., Baciu C., (2025). Cancer Prevalence across Vertebrates. Cancer Discov 15, 227–244. 10.1158/2159-8290.CD-24-0573. 39445720 PMC11726020

[24] Lee B., Oh S., Lee S., Kim Y., Youn S., Kim Y., Kwon S., and Kim D. (2015). Squamous cell carcinoma in a nine-banded armadillo (dasypus novemcinctus). J Zoo Wildl Med 46, 333–334. 10.1638/2013-0258R1.1. 26056888

[25] Pence D.B., Tran R.M., Bishop M.L., and Foster S.H. (1983). Fibroma in a Nine-banded armadillo (Dasypus novemcinctus). J Comp Pathol 93, 179–184. 10.1016/0021-9975(83)90004-x. 6863608

[26] Souto E.P.F., Oliveira A.M., Cardoso D.F., Figueiredo L.W.P., Kommers G.D., Galiza G.J.N., Mota R.A., and Dantas A.F.M. (2023). Osteosarcoma in a free-living yellow armadillo (Euphractus sexcinctus). J Comp Pathol 206, 9–12. 10.1016/j.jcpa.2023.08.003. 37717411

[27] Alves A. de D.F., Siqueira D.B. de, Rameh-de-Albuquerque L.C., Silva M.A., Pereira M. de F., Oliveira A.A. da F., and Silva Junior V.A. da (2018). Carcinoma mamário com metástase pulmonar em tatu-peba (Eupharactus sexcinctus). Acta sci. vet. (Impr.), 1–5.

[28] Navas-Suárez P.E., Sacristán C., Kluyber D., Yogui D.R., Alves A.C., Dalazen G.T., Díaz-Delgado J., Guerra J.M., de Azevedo Fernandes N.C.C., Réssio R.A., (2022). Novel gammaherpesvirus associated with primary gastric T-cell lymphoma in a free-ranging giant armadillo in Brazil. Transbound Emerg Dis 69, 2045–2051. 10.1111/tbed.14189. 34132049

[29] Marin-Padilla M., and Benirschke K. (1963). Thalidomide induced alterations in the blastocyst and placenta of the armadillo, dasypus novemcinctus mexicanus, including a choriocarcinoma. Am J Pathol 43, 999–1016. 14099460 PMC1949780

[30] Wang X., Xu X., An F., Ren Z., Li Y., Wang K., and Hua Y. (2024). Infantile hemangioma in a subadult Chinese pangolin: a case report. BMC Vet Res 20, 31. 10.1186/s12917-023-03832-9. 38267947 PMC10807128

[31] Chong S.M., Heng Y., and Yeong C.Y.-F. (2021). Pyelonephritis and Cystic Endometrial Hyperplasia in a Captive Sunda Pangolin (*Manis javanica*). Journal of Comparative Pathology 184, 101–105. 10.1016/j.jcpa.2021.03.003. 33894871

[32] Khatri-Chhetri R., Chang T.-C., Khatri-Chhetri N., Huang Y.-L., Pei K.J.-C., and Wu H.-Y. (2017). A retrospective study of pathological findings in endangered formosan pangolins (manis pentadactyla pentadactyla) from southeastern taiwan. Taiwan Veterinary Journal. 10.1142/S1682648515500316.

[33] Chu P.-Y., Zhuo Y.-X., Wang F.-I., Jeng C.-R., Pang V., Chang P.-H., Chin S.-C., and Liu C.-H. (2012). Spontaneous neoplasms in zoo mammals, birds, and reptiles in Taiwan – a 10-year survey in: Animal Biology Volume 62 Issue 1 (2012). Animal Biology 62, 95–110.

[34] Pseudo Dollo models for the evolution of binary characters along a tree | bioRxiv https://www.biorxiv.org/content/10.1101/207571v1.full.

[35] Neureiter N., Ranacher P., Efrat-Kowalsky N., Kaiping G.A., Weibel R., Widmer P., and Bouckaert R.R. (2022). Detecting contact in language trees: a Bayesian phylogenetic model with horizontal transfer. Humanit Soc Sci Commun 9, 1–14. 10.1057/s41599-022-01211-7.

[36] Ranwez V., Douzery E.J.P., Cambon C., Chantret N., and Delsuc F. (2018). MACSE v2: Toolkit for the Alignment of Coding Sequences Accounting for Frameshifts and Stop Codons. Mol Biol Evol 35, 2582–2584. 10.1093/molbev/msy159. 30165589 PMC6188553

[37] Kearse M., Moir R., Wilson A., Stones-Havas S., Cheung M., Sturrock S., Buxton S., Cooper A., Markowitz S., Duran C., (2012). Geneious Basic: an integrated and extendable desktop software platform for the organization and analysis of sequence data. Bioinformatics 28, 1647–1649. 10.1093/bioinformatics/bts199. 22543367 PMC3371832

