# Supplementary material for: Loss of the cytosolic DNA-sensing genes CGAS and STING1 in armadillos (Cingulata)
Source: bioRxiv. 2025 May 16:2025.05.13.651073. Preprint. [Version 1] doi: 10.1101/2025.05.13.651073 (PMC12132585; doi:10.1101/2025.05.13.651073)
Supplement: Supplement 1 [file NIHPP2025.05.13.651073v1-supplement-1.pdf]

bioRxiv preprint doi: https://doi.org/10.1101/2025.05.13.651073; this version posted May 16, 2025. The copyright holder for this preprint (which was not certified by peer review) is the author/funder, who has granted bioRxiv a license to display the preprint in perpetuity. It is made available under aCC-BY 4.0 International license.

Table S1: Details of xenarthran genome assemblies used.

Assembly Accession	Assembly Name	Species	Assembly Level	Release Date	Number of Scaffolds
GCF_030445035.2	mDasNov1.1.hap2	*Dasypus mexicanus*	Chromosome	13/07/2023	545
GCF_015220235.1	mChoDid1.pri	*Choloepus didactylus*	Chromosome	03/11/2020	145
GCA_023851605.1	mTamTet1.pri	*Tamandua tetradactyla*	Chromosome	22/06/2022	200
GCA_963992745.1	mBraTor.pri	*Bradypus torquatus*	Chromosome	06/12/2024	2,915
GCA_026826555.1	mTolMat1	*Tolypeutes matacus*	Scaffold	12/12/2022	54,571
GCA_000164785.2	C_hoffmanni-2.0.1	*Choloepus hoffmanni*	Scaffold	30/09/2014	269,084
GCA_004026745.1	MyrTri_v1_BIUU	*Myrmecophaga tridactyla*	Scaffold	16/01/2019	1,621,408
GCA_004027775.1	BraVar_v1_BIUU	*Bradypus variegatus*	Scaffold	16/01/2019	1,771,805
GCA_004027955.1	ChaVel_v1_BIUU	*Chaetophractus vellerosus*	Scaffold	16/01/2019	7,677,333
GCA_029593785.1	MVZ_CabUni_1.0	*Cabassous unicinctus*	Scaffold	05/04/2023	4,767,583
GCA_015220265.1	mChoDid1.alt	*Choloepus didactylus*	Scaffold	02/11/2020	11,980
GCA_004027855.1	ChoDid_v1_BIUU	*Choloepus didactylus*	Scaffold	16/01/2019	2,532,333
GCA_004025105.1	TamTet_v1_BIUU	*Tamandua tetradactyla*	Scaffold	15/01/2019	2,144,204
GCA_004025125.1	TolMat_v1_BIUU	*Tolypeutes matacus*	Scaffold	15/01/2019	2,925,507
GCF_000208655.3	Dasnov3.2	*Dasypus mexicanus*	Scaffold	06/01/2012	46,379
JBMPIZ000000000	BraTri_v1_ISEM	*Bradypus tridactylus*	Scaffold	Pending	15,237
JBMPJB000000000	CycDid_v1_ISEM	*Cyclopes didactylus*	Scaffold	Pending	14,869
JBMPJD000000000	MyrTri_v2_ISEM_DNAZoo	*Myrmecophaga tridactyla*	Scaffold	Pending	5,551
JBMPJF000000000	TamTet_v2_ISEM_DNAZoo	*Tamandua tetradactyla*	Scaffold	Pending	5,841
JBMPJC000000000	EupSex_v1_ISEM	*Euphractus sexcinctus*	Scaffold	Pending	10,534
JBMPJA000000000	ChlTru_v1_CT01	*Chlamyphorus truncatus*	Scaffold	Pending	36,203
JBMPJE000000000	PriMax_v1_M844	*Priodontes maximus*	Scaffold	Pending	51,157
